# Sim-to-real *via* latent prediction: Transferring visual non-prehensile manipulation policies

**DOI:** 10.3389/frobt.2022.1067502

**Published:** 2023-01-12

**Authors:** Carlo Rizzardo, Fei Chen, Darwin Caldwell

**Affiliations:** ^1^ Active Perception and Robot Interactive Learning Laboratory, Advanced Robotics, Istituto Italiano di Tecnologia, Genova, Italy; ^2^ Department of Mechanical and Automation Engineering, T-Stone Robotics Institute, The Chinese University of Hong Kong, Hong Kong, China

**Keywords:** reinforcement learning (RL), robotics, manipulation, variational techniques, dynamics, pushing

## Abstract

Reinforcement Learning has been shown to have a great potential for robotics. It demonstrated the capability to solve complex manipulation and locomotion tasks, even by learning end-to-end policies that operate directly on visual input, removing the need for custom perception systems. However, for practical robotics applications, its scarce sample efficiency, the need for huge amounts of resources, data, and computation time can be an insurmountable obstacle. One potential solution to this sample efficiency issue is the use of simulated environments. However, the discrepancy in visual and physical characteristics between reality and simulation, namely the sim-to-real gap, often significantly reduces the real-world performance of policies trained within a simulator. In this work we propose a sim-to-real technique that trains a Soft-Actor Critic agent together with a decoupled feature extractor and a latent-space dynamics model. The decoupled nature of the method allows to independently perform the sim-to-real transfer of feature extractor and control policy, and the presence of the dynamics model acts as a constraint on the latent representation when finetuning the feature extractor on real-world data. We show how this architecture can allow the transfer of a trained agent from simulation to reality without retraining or finetuning the control policy, but using real-world data only for adapting the feature extractor. By avoiding training the control policy in the real domain we overcome the need to apply Reinforcement Learning on real-world data, instead, we only focus on the unsupervised training of the feature extractor, considerably reducing real-world experience collection requirements. We evaluate the method on sim-to-sim and sim-to-real transfer of a policy for table-top robotic object pushing. We demonstrate how the method is capable of adapting to considerable variations in the task observations, such as changes in point-of-view, colors, and lighting, all while substantially reducing the training time with respect to policies trained directly in the real.

## 1 Introduction

To this day, manipulation and physical interaction tasks remain open problems in robotics. The difficulty of modeling the environment, identifying its characteristics, and detecting and tracking elements of interests makes these tasks particularly challenging for classic control approaches. Reinforcement Learning approaches instead can tackle these issues implicitly, and have been shown to be capable of solving even the most complex manipulation problems OpenAI et al. (2019). However, the use of Reinforcement Learning (RL) methods also poses significant challenges. Most RL techniques are considerably sample inefficient, they require huge amounts of resources, data and computation time. Also, training a policy on real hardware without proper precautions may damage the hardware itself or its surroundings. These issues can be tackled from different perspectives, on one side with algorithmic improvements that improve sample efficiency and lower data requirements, on the other with techniques to efficiently acquire huge amounts of data, for example by exploiting simulation [OpenAI et al. (2019); Rudin et al. (2021)].

Standard RL algorithms such as DQN Mnih et al. (2015), PPO Schulman et al. (2017), or SAC (Haarnoja et al. (2018a); Haarnoja et al. (2018b)) have huge data requirements, especially for vision-based tasks. Such tasks have traditionally been solved by directly utilizing image observations in an end-to-end manner, the same way as tasks with low-dimensional observations are handled. Several recent works however have progressively improved sample efficiency for visual tasks by departing from this simple approach.

SAC-AE [Yarats et al. (2021b)], SLAC [Lee et al. (2020)] or CURL Srinivas et al. (2020) have tackled the problem by combining Reinforcement Learning and Representation Learning methods. Representation Learning is used to aid the training of the visual feature extractor section of the agent. In purely RL methods, the training is performed solely from the reward signal, even for what concerns visual understanding. Here instead other sources of information are used such as image reconstruction or contrastive losses, greatly improving sample efficiency.

Other approaches, such as RAD [Laskin et al. (2020a)] and DrQ [Kostrikov et al. (2020); Yarats et al. (2022)] have shown how the use of simple image augmentation techniques can vastly improve sample efficiency, reaching performance on par with that of methods which have access to state knowledge.

Another direction yet has been the idea of using the experience data collected online during training to learn a model of the environment, capable of predicting whole trajectories. These kind of models can then be used to solve the task *via* planning, like for example in PlaNet [Hafner et al. (2019)], or to generate additional training data, either in the observation space or in learned latent spaces, such as in Dreamer [Hafner et al. (2020); Hafner et al. (2021)].

In this work we explore the idea of exploiting Representation Learning and environment modeling to efficiently perform sim-to-real transfer. We define an RL agent that completely decouples feature extractor and control policy training. The feature extractor is learned as part of a full model of the environment based on Variational Autoencoders (VAE) [Kingma and Welling (2014); Rezende et al. (2014)], capable of predicting observations and rewards. The control policy is a Soft Actor-Critic agent that acts on the latent representation defined by the aforementioned model. We show how this architecture allows to transfer a control policy trained in simulation to the real world by only finetuning the encoder and decoder sections of the VAE model. This completely removes the need of performing Reinforcement Learning training in the real environment, strongly reducing real-world data requirements while at the same time maintaining high sample efficiency in simulation.

We evaluate the method on a tabletop non-prehensile manipulation task, in which a Franka-Emika Panda robotic arm has the objective of pushing an object to a predetermined destination. We choose this task as it is fairly simple and manageable, but at the same time presents difficulties that make it a suitable ground for evaluating model-free reinforcement learning methods such as ours. As discussed in Ruggiero et al. (2018), non-prehensile manipulation, and specifically object pushing, is a particularly challenging task for classic control methods due to the indeterminacy brought by friction forces, both between manipulated object and ground and between object and robot. Modeling such interactions precisely is extremely challenging, identifying friction characteristics is a complex problem in itself and minute errors in the modeling have large impacts in the motion of the manipulated objects. Instead, model-free robot learning approaches such as ours handle these problematics implicitly without requiring careful explicit modeling of the system and can consequently solve this task effectively and reliably.^1^ Also, object pushing already presents exploration difficulties not present in simpler tasks, such as for example pose reaching. The agent has to first learn to reach the object, and then it must learn to bring it to the correct position. We perform sim-to-sim experiments with different variations of the scenario, from simple alterations to the colors of the scene to radical changes in the camera point of view. We then validate the approach with sim-to-real experiments.

## 2 Related works

Our work builds upon the intersection of two research areas: the use of sim-to-real to overcome real-world data scarcity and the development of decoupled Reinforcement Learning methods. The first focuses on exploiting simulation data to train real-world models by bridging the reality gap, the second on improving sample efficiency in Reinforcement Learning, by decoupling feature extraction from policy training.

The RL architecture we propose exploits its decoupled nature to effectively overcome the reality gap, reducing real-world data requirements while at the same time maintaining good sample efficiency in the simulation domain.

### 2.1 Sim-to-real

Reinforcement Learning methods require vast amounts of data to be effectively trained. The more a task is complex, in terms of observation and action dimensionality, or exploration difficulty, the more experience is required. Complex tasks can easily require days or weeks of experience data to be solved. Acquiring such amounts of experience on real robotic systems is impractical, keeping a complex robotic system running for such lengths of time is complex, additional infrastructure for managing the environment setup are required, and untrained policies can potentially damage the robot or the environment. All of these issues become even more complex in a research environment, where numerous trainings have to be performed for experimental reasons. Consequently, the use of synthetic experience has a natural appeal for Reinforcement Learning methods.

Sim-to-real RL methods exploit simulation software to efficiently train policies in virtual reproductions of the target environment, and then transfer the policy to the real-world domain by overcoming the reality gap, the discrepancy between simulation and reality.

The advantage of simulation is first of all the possibility of generating vast amounts of experience much more rapidly than it would be possible in the real world. This can be achieved by simulating faster than real-time and by parallelizing multiple simulated environments. Gorila Nair et al. (2015), A2C and A3C Mnih et al. (2016) showed how parallelizing experience collection leads to substantial improvements in training time. More recently, Rudin et al. (2021) exploited modern hardware and simulation software to massively parallelize an environment for quadruped locomotion training, achieving in just 20 min a PPO gait policy capable of successfully controlling a real robot on complex terrains.

Furthermore, beyond just generating huge amounts of data, simulation software can also support training strategies that would be impossible in the real world. Pinto et al. (2018) shows how it is possible to speed-up training considerably by using simulator state knowledge during training, and how to transfer a policy trained in such a way to the real world, where this knowledge is unavailable.

As we mentioned, the core issue with simulation training is the reality gap, the discrepancy between the characteristics of the simulated environment and those of the real one. These differences can be in the dynamics of the environment, due to inaccuracies in the physics simulation, in the observations the agent makes, due to imprecision in the visual rendering or in the sensory input in general, or simply in the behavior of robotic components, which may be implemented differently in simulation and reality. Advances in realistic simulation software [NVIDIA (2020); Unity (2020)] are progressively narrowing the reality gap, but sim-to-real transfer remains non-trivial as constructing simulations that closely match the real world remains a challenging task that requires considerable engineering work.

Numerous strategies have been implemented to overcome the reality gap. In general, we can distinguish between two families of techniques: those that aim at obtaining a policy capable of operating in both the real and the simulation without using real-world data, and those that use real-world data for adapting a model learned in simulation to the real domain. We refer to these latter ones as *domain adaptation* methods. The most simple approach of these is to just perform policy finetuning in the real, the same way it usually is done in supervised learning settings. The policy is first trained in simulation, then the agent is transferred to the real and the training continues in the real until satisfactory performance is achieved. However, such strategy often still requires considerable real-world experience collection, and it is not guaranteed the robot will behave properly and safely when first transferred to the real domain.

Other methods explicitly target the issue of matching the output of feature extractors between the simulated domain and the real domain, creating feature extractors that are invariant to the switch between simulated and real-world inputs. This can be achieved *via* different approaches. Some methods try to train feature extractors for the two domains while keeping the distributions of the two resulting feature representations similar, with losses based on distribution distance metrics such as Maximum Mean Discrepancy (MMD) [Tzeng et al. (2014)], MK-MMD [Long et al. (2015)] or others [Sun and Saenko (2016)]. Others try to keep the feature representations of samples from the two domains close *via* Adversarial approaches. A discriminator network is trained to classify feature vectors between the two domains, the feature extractor is then optimized to generate indistinguishable representations [Tzeng et al. (2015); Tzeng et al. (2017); Ganin and Lempitsky (2015)]. Alternatively, other techniques take inspiration from style transfer methods and directly convert target-domain samples into source-domain samples or samples from a third “canonical” domain [Bousmalis et al. (2017); Bousmalis et al. (2018); Hoffman et al. (2018); James et al. (2019)]. Other methods attempt to identify corresponding samples from source and target domain and then force the representations of these corresponding samples to be similar. Gupta et al. (2017) does so by assuming samples from corresponding timesteps in RL episodes should be similar, Tzeng et al. (2016) first identifies weakly paired samples and improves on this with an adversarial approach.

However, even if some of these methods work well for vision tasks, they may not adapt effectively to difficult exploration problems. The aforementioned approaches either require target data to be available while performing the original source domain training or they train the encoder with offline data. This is problematic, as in difficult exploration problems collecting fully representative data before completely training the policy may be impractical or impossible. In tasks such as object pushing it may be possible to collect human-generated demonstrations, but in more complex tasks, for example locomotion, collecting demonstrations is not trivial.

A sim-to-real approach that does not suffer from this issue is *Domain Randomization*. The core idea of the method is to randomize visual [Tobin et al. (2017)] and physical [Peng et al. (2017)] characteristics of the simulated environment, so that once the agent is transferred in the real world it can interpret the new domain as just another random variation. These methods can be applied to both visual and state-based tasks, and have been extremely successful, being able to solve even extremely complex visuomotor control problems while maintaining strong robustness to occulusions and perturbations [OpenAI et al. (2019)]. However, as tasks get more complex, they require huge amounts of simulation data and long training times. To reduce these issues, various methods have been proposed to constrain the amount of randomization to just what is necessary. Ramos et al. (2019), Possas et al. (2020) and Muratore et al. (2021) achieve this by identifying simulator parameter distributions *via* Likelihood-free Inference. Heiden et al. (2021) instead shows how it is possible to use differentiable simulators to identify possible simulator parameters from real data.

### 2.2 Decoupled RL

Training Reinforcement Learning policies for visual tasks has traditionally been considerably more expensive than for state-based tasks, in terms of sample-complexity and computation time. The increased dimensionality of the observation space naturally complicates the problem, as the agent needs to learn to interpret visual information and extract the necessary features from it. However, multiple recent works have shown how the performance gap between image-based and state-based tasks can be greatly reduced.

One extremely simple and effective technique is the use of data augmentation during training. RAD [Laskin et al. (2020a)], DrQ [Kostrikov et al. (2020)] and DrQv2 [Yarats et al. (2022)] have shown how even just simple image augmentations such as pixel shift can drastically improve sample efficiency of model free RL methods, reaching performance comparable to that achieved on equivalent state-based tasks.

Other works instead exploit unsupervised learning methods to aid the extraction of visual features. SLAC [Lee et al. (2020)] trains a predictive stochastic latent variable model and uses the resulting latent space to train a Soft Actor-Critic policy. SAC + AE (Yarats et al. (2021b)) instead uses a regularized autoencoder [Ghosh et al. (2020)] to extract a latent space *via* observation reconstruction. It then uses the resulting latent vector as inputs for a Soft Actor-Critic policy.

PlaNet (Hafner et al. (2019)) brings these ideas forward by learning a full latent model of the environment, then uses this model to plan trajectories *via* Model Predictive Control. Dreamer (Hafner et al. (2020)) and DreamerV2 [Hafner et al. (2021)] then use the latent model from PlaNet to train an Actor-Critic policy in the latent space, generating huge amounts of experience *via* imagination and exploiting the differentiable nature of the neural network model.

## 3 Methods

The method we propose in this work employs ideas from research in decoupled Reinforcement Learning methods to perform sim-to-real transfer of visuomotor control policies *via* domain adaptation. More specifically we define an RL architecture composed of a predictive latent variable model and a Soft Actor-Critic agent, and we propose a training procedure for sim-to-real that takes advantage of the presence of the decoupled predictive model to effectively finetune the latent encoder to the real environment. Following this method it is possible to transfer a trained agent from simulation to reality by finetuning just the feature extractor, independently of the learned control policy.

Differently from the methods discussed in Section 2, our proposed method is at the same time unsupervised, sample efficient in source and target domain, capable of solving difficult exploration problems, and does not require target domain data before performing the transfer.

### 3.1 Problem formulation

**FIGURE 1:**
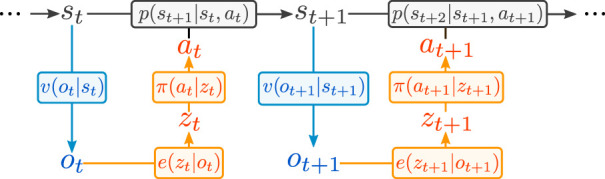
POMDP formulation of the problem. In orange the policy we implement, in blue the sensory channel, in black the underlying Markov Decision Process.

We formalize our setting as a *Partially Observable Markov Decision Process* (POMDP), defined by the 7-tuple (*S*, *A*, *p*, *r*, *γ*, *O*, *v*). The first five terms represent respectively the state space, the action space, the state transition probability density *p*(*s*_*t*+1_|*s*_*t*_, *a*_*t*_), the reward function *r*(*s*_*t*_, *a*_*t*_) and the discount factor. The last two terms represent the observation space and observation density *v*(*o*_*t*_|*s*_*t*_) = *p*[*o*_*t*_ = *o*|*s*_*t*_ = *s*] which defines our sensory channel. The system is represented in Figure 1.

The objective of the method is to learn a policy *π*(*a*_*t*_|*o*_*t*_) that maximizes the expected discounted total sum of rewards $R\left(\pi \right)=E_{s_{0:T}}\left(\sum_{t=0}^{T}\gamma^{t}r\left(s_{t},a_{t}\right)\right)$ .

Historically, standard end-to-end RL methods have usually employed an MDP formulation instead of a POMDP one, meaning that the state is observed directly or the high-dimensional observation is equated with the state. Instead, in the POMDP formulation the state is not observed directly, but only through a stochastic sensory channel, formalized with the *v*(*o*_*t*_|*s*_*t*_) density function.

Guided by this formulation, we use a representation learning approach to approximate an *e*(*z*_*t*_|*o*_*t*_) density, which estimates a state representation *z*_*t*_ from sensor observations *o*_*t*_. Once the representation is learned, we have at our disposal a method to approximate low-dimensional state vectors from high-dimensional sensor inputs and we can use standard RL methods to learn a control policy in the latent space.

### 3.2 Agent architecture

#### 3.2.1 Variational autoencoder

A natural choice for learning the state representation is the use of *autoencoders*, in particular of *variational autoencoders* (VAE) Kingma and Welling (2014). VAEs are a solid and proven method to learn a low-dimensional representation of the state, giving us a method to reliably produce low-dimensional latent vectors from high-dimensional observations. We define our VAE architecture as a stochastic encoder *e*_*θ*_(*z*_*t*_|*o*_*t*_) that maps observations $o_{t}\in R^{n\times n}$ to latent representations $z_{t}\in R^{k}$ and a deterministic decoder *d*_*θ*_(*z*_*t*_) that performs the opposite transformation. The encoder is defined as a conditional multivariate Gaussian density with diagonal covariance. The dimensionality *k* of the latent space is left as a hyperparameter, which can be tuned depending on the task at hand.

In all our experiments we used an encoder architecture based on a MobileNet V3 backend Howard et al. (2019) initialized with pretrained weights, the output of the backend was matched to the Gaussian density mean and log-variance with two separate linear layers. The decoder was defined symmetrically using transposed convolution layers.

In practice, during policy inference we used the encoder deterministically by utilizing only the mean of the distribution to produce the latent vectors.

#### 3.2.2 Dynamics modeling

Within the latent space of the Variational Autoencoder we introduce a one-step dynamics predictor, that from a latent vector and an action predicts a latent representation for the next state. Formally, the dynamics predictor is defined as a function $f\left(z_{t},a_{t}\right)\mapsto {\hat{z}}_{t+1}$ , where *z*_*t*_ is the latent representation for the state *s*_*t*_, *a*_*t*_ is the action at time *t* and ${\hat{z}}_{t+1}$ is a representation of *s*_*t*+1_. In practice the dynamics model is implemented as a fully connected neural network $f_{\theta }:\;R^{k+m}\to R^{k}$ with *m* being the action space dimensionality and *k* being the latent representation size. We make the choice of introducing the predictor following two intuitions: one is that the presence of the dynamics predictor imposes a regularization toward features more suited for control, the other is that the presence of the predictor can be used to constrain the latent representation when performing policy transfer. We refer to this overall representation architecture as DVAE.

It must be noted that, while our dynamics model is useful in shaping and constraining the latent representation, it cannot be used to make actual latent-space trajectory predictions. This is because the input and output latent spaces of the network are not constrained to represent the same features in the same way, or to have the same distribution.

As a whole, the architecture is trained using the usual variational loss, composed of the isotropic multivariate Gaussian KL-divergence and the MSE reconstruction error:

$$\begin{array}{c} L_{\mathrm{DV AE}}\left(\theta ;o_{t},a_{t},r_{t},o_{t+1}\right)=D_{KL}\left(e_{\theta }\left(z_{t}|o_{t}\right)\|N\left(0,I_{k}\right)\right)\\ \;+\;\alpha MSE\left({\hat{o}}_{t+1},{\hat{r}}_{t};o_{t+1},r_{t}\right) \end{array}$$ (1)

With $\left({\hat{z}}_{t+1},{\hat{r}}_{t}\right)=f_{\theta }\left(e_{\theta }\left(o_{t}\right),a_{t}\right)$ and ${\hat{o}}_{t+1}=d_{\theta }\left({\hat{z}}_{t+1}\right)$ , *D*_*KL*_ being the KL-divergence, *MSE* the mean squared error, *I*_*k*_ the *k* × *k* identity matrix and $N\left(0,I_{k}\right)$ a centered isotropic Gaussian distribution.

#### 3.2.3 Including low-dimensional sensor data

As we mentioned, the encoder and decoder sections of the architecture are implemented respectively with a MobileNet network and a series of transposed convolutions. These architectures are suited to image inputs, however, in robotics applications it is common to have non-homogeneous sensory inputs, some characterized by a high dimensionality, like cameras, and others by low-dimensionality, like motor encoders or force torque sensors. Our proposed architecture gives us a natural way to combine these heterogeneous inputs, as we can combine all of these observations of the environment into the latent state representation.

In the simple case of one visual input combined with mono-dimensional sensor readings, we can leave the encoder architecture unchanged and directly concatenate the low-dimensional observations to the encoder output. For its simplicity, we chose to follow this simple approach in our experiments.

**FIGURE 2:**
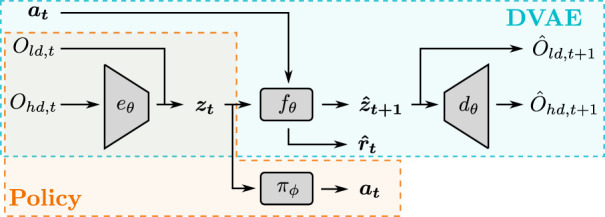
Overview of the proposed architecture. *O*_*hd*,*t*_ indicates the high-dimensional observation at step *t*, *O*_*ld*,*t*_ is the respective low-dimensional observation, *a*_*t*_ is the action taken at step *t*, *z*_*t*_ is the latent state vector at time *t*, *z*_*t*+1_ is the predicted latent state vector, *O*_*hd*,*t*+1_ and *O*_*ld*,*t*+1_ are the predicted observations.

The overall architecture is represented in Figure 2.

#### 3.2.4 Policy learning

Finally, the control policy *π*(*a*_*t*_|*o*_*t*_) can be learned with any standard RL method. The RL algorithm only receives as input the state representation *z*, composed of the encoder output and, optionally, the low-dimensional observations. In practice we chose to use *Soft Actor Critic* (SAC) throughout our experiments because of the flexibility and generality of the method. We derived our implementation from the one provided by *stable_baselines3* Raffin et al. (2021).

#### 3.2.5 Bootstrap ensembles

To improve the reliability and repeatability of the method, and following evidence from Chua et al. (2018) and Nagabandi et al. (2019) we exploit *bootstrap ensembles* in the dynamics model, in the encoder and in the SAC actor network. The output of the single networks are aggregated performing a simple average. This in practice results in a more reliable training performance, converging faster to a correct solution and reducing the variability introduced by the network initialization and the environment randomness.

### 3.3 Training the agent

**FIGURE 3:**
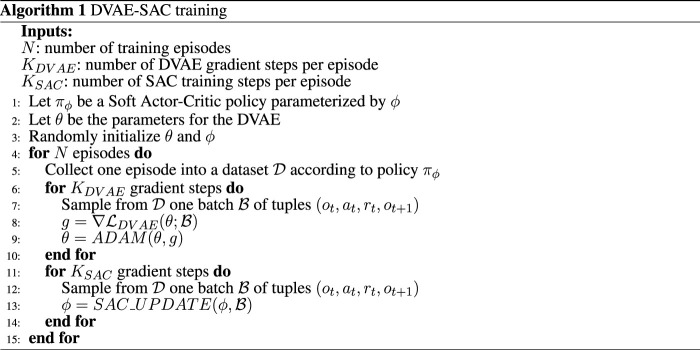
Training procedure for DVAE-SAC. DVAE weights are updated with ADAM Kingma and Ba (2015) and the policy is trained with SAC_UPDATE as defined in Haarnoja et al. (2018b).

We train the DVAE-SAC agent online, by collecting experience *via* the current policy and alternating between training the DVAE latent extractor and the SAC control policy. The experience is collected in the form of (*o*_*t*_, *a*_*t*_, *r*_*t*_, *o*_*t*+1_) transitions and stored in one single replay buffer, which is used as the training set for both the policy and the latent extractor. Algorithm 1 at Figure 3 shows the overall training procedure.

### 3.4 Transferring the agent

In performing the domain transfer the objective is to adapt the agent to the new environment while avoiding to lose the knowledge acquired during the source domain training. Such transfer could be attempted by simply finetuning the whole agent on target domain data, however in practice this does not perform well due to catastrophic forgetting. This is particularly problematic as policy training may potentially restart from scratch, as no experience for the later stages of successful episodes would be available until the environment is explored again.

To prevent these issues, we take advantage of the decoupled nature of the DVAE-SAC architecture: we freeze the SAC agent and transfer it as-is, while only finetuning the DVAE. Crucially, to prevent the latent representation from drifting and becoming incompatible with the SAC policy we also freeze the dynamics predictor section of the DVAE. In practice this means only the encoder and decoder sections of the architecture are adapted to the target domain.

By keeping the dynamics predictor frozen the DVAE is constrained to maintain a latent representation compatible with the dynamics predictor itself. We show experimentally that this is sufficient for the policy to keep operating correctly, as compatibility with the policy is also maintained.

## 4 Experiments

**FIGURE 4:**
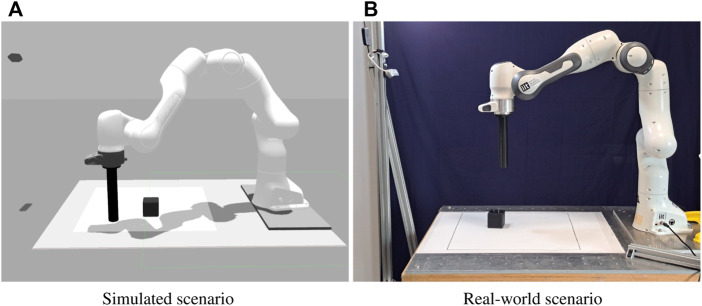
Simulated **(A)** and real **(B)** object pushing setups. The camera used to collect the input images is visible in the top left in both pictures.

**FIGURE 5:**
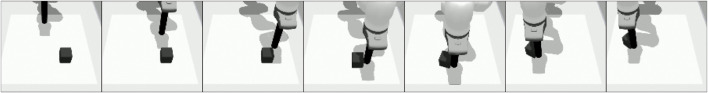
Example of one successful episode in the simulated setup. The image shows the observed images for steps 0, 4, 8, 12, 16, 20, 24.

To demonstrate the effectiveness of the method we evaluate its performance on a robotic table-top object pushing task. In our scenario a 7-DOF Franka Emika Panda robotic arm is tasked with pushing a 6 cm cube to a target position. The robot arm is controlled in cartesian space, and the end-effector moves only horizontally within a 45 cm square workspace located in front of the robot itself. Each episode is initialized with a random cube position and a random end-effector position. The target cube position is kept constant across episodes. The agent controls the robot specifying a displacement in the bidimensional workspace of the end-effector, resulting in a continuous 2D action space. The environment is observed through a camera placed on the opposite side of the table with respect to the robot arm, which produces RGB images with 128 × 128 pixels resolution. In addition to the images the agent also has access to proprioceptive information from the robot, in the form of the 2D position of the end-effector tip. Table 1 summarizes the observation and action spaces. Figure 4 shows the simulated and real scenarios, Figure 5 displays an example of a successful episode.

**TABLE 1:** Object pushing environment observation and action spaces. The action space is normalized to [−1, 1], but corresponds to a displacement of maximum 2.5 cm in *x* and *y*.

Observation space	[0,1]^128 × 128^ × [−1,1]^2^
Action space	[−1,1]^2^

Each episode lasts 40 steps. Once the cube reaches the target position, within a 5 cm tolerance, the episode is considered successful, but it is not interrupted until the 40 steps timeout is reached.

We define the reward function as a composition of three terms, one to encourage the end-effector tip to stay close to the cube, one for the cube to stay close to the goal, one for the cube to be moved in any direction. We define them as follows, where *r*(*p*_*c*_, *p*_*t*_) is the overall reward, *r*_*c*_(*p*_*c*_, *p*_*g*_) is the cube-goal term, *r*_*t*_(*p*_*t*_, *p*_*c*_) the tip-cube term, $r_{d}\left(p_{c},p_{c}^{\prime }\right)$ the cube displacement term, and *r*_*b*_(*p*_*c*_, *p*_*g*_) is a further bonus given when the cube is within *d* m from the target. The constant *α* is a scaling factor, which we kept fixed at .1. The *r*_*c*_ and *r*_*t*_ functions are defined as linear ramps, with value 0 at 40 cm from the target and respectively 100 and 50 at the target.

$$\begin{array}{cc} & r\left(p_{c},p_{t}\right)=\alpha *\left(r_{c}\left(p_{c},p_{t}\right)+r_{t}\left(p_{t},p_{c}\right)+r_{d}\left(p_{c},p_{c}^{\prime }\right)\right)\\ \text{With:}\\ & r_{c}\left(p_{c},p_{g}\right)=\frac{100}{0.4}*\left(0.4-\|p_{c}-p_{g}\|\right)+r_{b}\left(p_{c},p_{g}\right)\\ & r_{t}\left(p_{t},p_{c}\right)=\frac{50}{0.4}*\left(0.4-\|p_{t}-p_{c}\|\right)\\ & r_{d}\left(p_{c},p_{c}^{\prime }\right)=\|p_{c}-p_{c}^{\prime }\|*100*20\\ & r_{b}\left(p_{c},p_{g}\right)=\left\{\begin{array}{c} \frac{200}{d}*\left(d-\|p_{c}-p_{g}\|\right)\;\text{ if }\|p_{c}-p_{g}\|\leq d\;\\ 0\;\text{otherwise}\; \end{array}\right. \end{array}$$ (2)

We implement this scenario both in the real world and in a *Gazebo* simulation [Koenig and Howard (2004)]. To evaluate transfer capability we perform both sim-to-sim and sim-to-real experiments, varying the characteristics of the simulation to enlarge or reduce the gap between source and target domains. Table 2 summarizes the characteristics of each scenario.

**TABLE 2:** Variations from the source domain across the different experimental scenarios.

Scenario	Cube color	Light direction	Camera position	Camera orientation
Original (Sim)	Black	Vertical	∖	∖
S2S—Minimal Gap	10 colors	Vertical	Unchanged	Unchanged
S2S—Small Gap	Red	30°: Left, Right, Back, Front	∼5 cm offset	Unchanged
S2S—Medium Gap	Red	30°: Left, Right, Back, Front	∼20 cm offset	Toward Center
S2S—Large Gap	Red	30° Left	∼70 cm offset	90° Yaw
S2R—Minimal Gap	Black	Multiple sources, diffused	∼5 cm offset	Minimal

### 4.1 Sim-to-sim

We evaluate our proposed method on four sim-to-sim transfer scenarios of increasing difficulty. We do so by keeping a fixed source domain and defining four sets of target domains, in which we vary the width of the transfer gap by altering characteristics such as the cube color, the lighting, and the camera pose.

**FIGURE 6:**
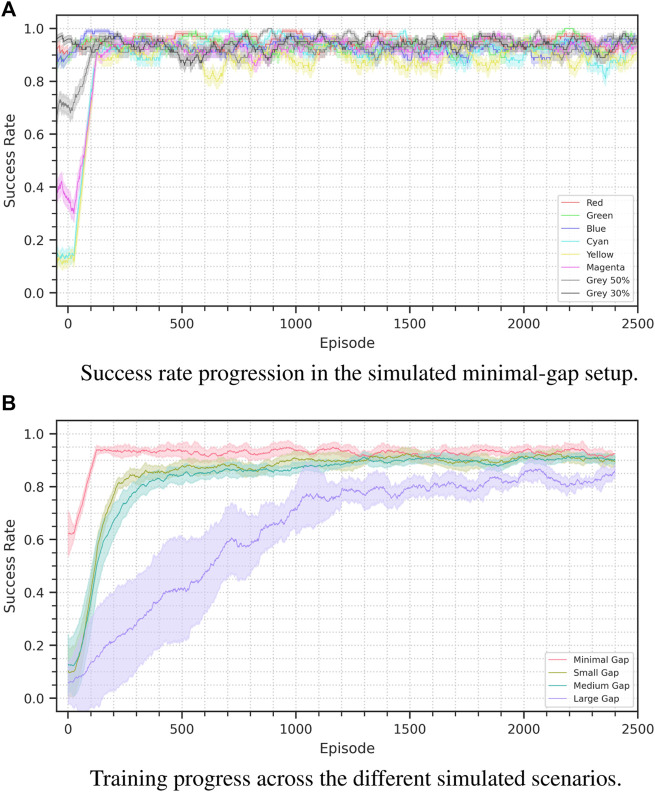
Success rate progress for the sim-to-sim experiments. Figure **(A)** shows the minimal-gap scenario: It is possible to see the performance difference depending on the cube color. Figure **(B)** displays the results for all the scenarios in an aggregated form, the plots show the average performance across seeds on a 100 episode window. For the minimal, small, medium and large scenarios we used respectively 8, 12, 16, and 4 seeds. The shaded area represents a 95% confidence interval.

**FIGURE 7:**
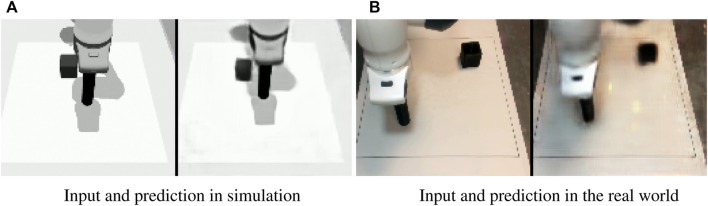
Input image and predicted image in the simulated **(A)** and real **(B)** setups.

In this section we discuss the different setups and the respective results. The results are also reported in Table 3 and Figure 6. Figure 7 shows the performance achieved training from scratch in simulation.

**TABLE 3:** DVAE-SAC results on the four sim-to-sim (S2S) and the sim-to-real (S2R) scenarios. Columns indicate respectively: the best achieved success rate, the initial success rate in the target domain (i.e. zero-shot transfer performance), the number of episodes required to reach 80% success rate, the number of episodes required to reach 90% success rate.

Scenario	Success (%)	Init. Succ (%)	T.T. 80%	T.T. 90%
Sim. From Scratch	98	5	1,020 Eps	1,180 Eps
S2S—Minimal Gap	95	50	90 Eps	110 Eps
S2S—Small Gap	92	5	210 Eps	950 Eps
S2S—Medium Gap	92	5	320 Eps	1,250 Eps
S2S—Large Gap	85	5	1,200 Eps	∖
S2R—Minimal Gap	92	5	550 Eps	990 Eps

#### 4.1.1 Minimal gap

In this scenario we only change the color of the manipulated cube. While in the source domain the cube is black, we define eight target scenarios with eight different colors: red, green, blue, yellow, cyan, magenta and 2 grades of gray.

Depending on the selected color the policy has an initial performance that varies between 10 and 95 percent, however in just 110 episodes of finetuning the method consistently reaches a 90% success rate, and then continues maintaining a performance oscillating between 92 and 95 percent.

#### 4.1.2 Small gap

In this setup we introduce variations also in the camera pose and the light direction. We vary the pose by translating it left, right, up or down of 5 cm. We vary the light direction from the vertical axis of the source domain to four possible axes with a 30° inclination either toward the left, right front or back. We set the cube color to be red.

With these variations the agent performance is initially of about 5%, which is comparable to a random policy. Across our experiment the agent consistently reaches an 80% success rate in about 210 episodes and a 90% success rate in about 950. However, already at episode 500 the agent consistently reaches an 88% success rate.

#### 4.1.3 Medium gap

To further widen the transfer gap in this scenario we increment the magnitude of the camera pose change. We move the camera of 20 cm instead of 5, and we alter its orientation to maintain the manipulation area in the field of view. We fix the cube color as red and vary light direction in the same way as in the previous scenario.

The training performance is comparable to that of the small-gap scenario, reaching 80% success rate in 320 episodes and 90% in 1,250. Also in this case performance just under 90% is reached considerably sooner, reaching 85% at episode 750.

#### 4.1.4 Large gap

In the hardest sim-to-sim scenario we completely change to camera point of view, while still altering cube color and light direction. The camera is moved so that it faces the manipulation area from the side instead of the front, with a 90° point of view change.

In this scenario, which goes beyond what is just a sim-to-real transfer problem, the agent training requires about as much time as is required to train the agent from scratch. About 1,200 episodes are required to reach an 80% success rate, and a maximum performance of 86% is reached by episode 2000, performing worse than the source training.

We hypotesize, this can be explained by the fact that the agent in this case encounters again an exploration problem, despite not using any kind of Reinforcement Learning method. The agent must again discover where the goal is located, and can only understand this from the training signal of the reward predictor present in the dynamics model.

### 4.2 Sim-to-sim with VAE-SAC

**FIGURE 8:**
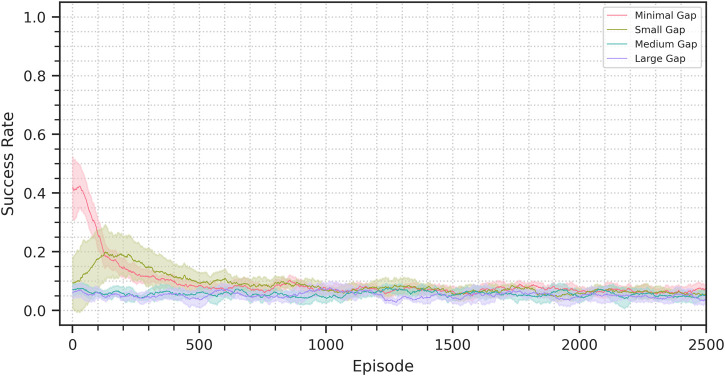
Success rate progress for the sim-to-sim VAE-SAC experiments. The plots show the average performance across seeds on a 100 episode window. The shaded area represents a 95% confidence interval.

To explore the importance of the dynamics predictor presence for domain transfer we evaluated the performance of a VAE-SAC agent on the sim-to-sim scenarios. The VAE-SAC agent is a modified version of our architecture in which the dynamics predictor has been removed. Figure 8 shows the achieved transfer performance. As expected the domain transfer fails, as there is no constraint to keep the latent representation compatible with the control policy. Even in the minimal-gap scenarios, where the zero-shot performance is not zero, the success rate rapidly descends to performance comparable to that of a randmo policy. In the small-gap scenario we can see the performance initially rising, but then also decaying.

### 4.3 Sim-to-real

**FIGURE 9:**
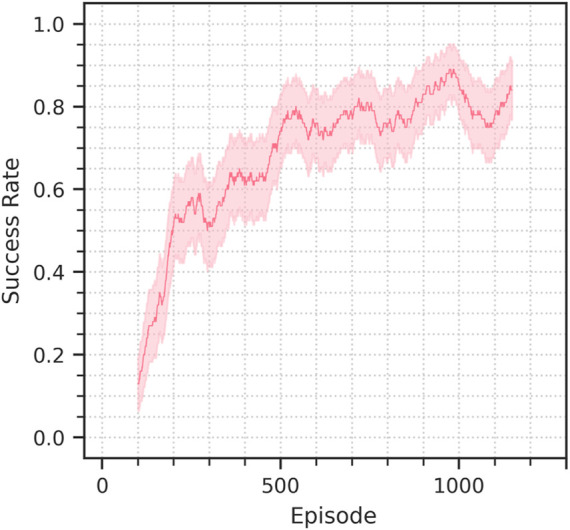
Success rate progression in the sim-to-real experiment discussed in section 4.3. The solid line represents the success rate in the 100-episode window preceding the current episode, the background bands represent the corresponding 95% confidence interval.

**FIGURE 10:**
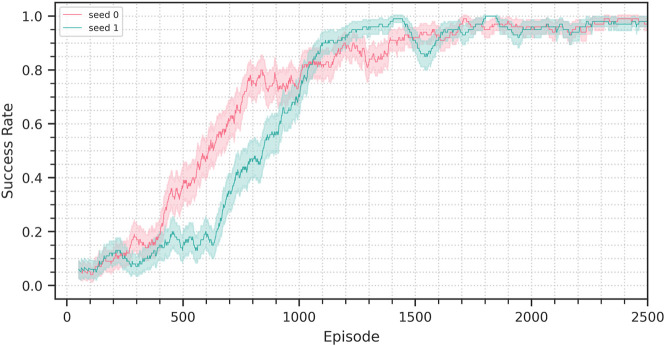
Success rate progression for the training from scratch performed in simulation. Two random seeds are being shown. The solid lines represents the success rate in the 100-episode window preceding the current episode, the background bands represent the corresponding 95% confidence interval.

In addition to the sim-to-sim evaluation we also assess the performance of the method on a sim-to-real transfer scenario. We only perform what we call a minimal-gap sim-to-real transfer, in which we minimize the differences by not intentionally introducing variations and trying to replicate the simulated scenario for what is possible. However, the transfer still presents small differences in the camera pose, and lighting and the texture of the environment is considerably different. Figure 9 shows the training performance in the real setup. Figure 10 shows the camera view and decoder reconstruction in the simulated and real scenarios.

The initial success rate achieved by the policy is about 10%, but the finetuning of the DVAE quickly brings it 80% in just 550 episodes, corresponding to 5 h of experience, a performance of 90% is achieved in 990 episodes, 10 h of experience data.

When compared to the training from scratch in simulation, the sim-to-real finetuning is considerably faster in the first stages of learning, achieving 80% success rate in about half the time, however reaching 90% requires almost as much time as the source training. It must be noted however that a training from scratch in the real requires considerably more time than in simulation, due to the higher complexity of the sensory inputs. Furthermore, the sim-to-real finetuning could be completed by unfreezing the control policy once the DVAE has reached good enough performance, allowing the agent to adapt to any further unaccounted difference in the target domain.

## 5 Conclusion

In this work we presented a method for efficiently learning visual manipulation policies and transferring trained agents from simulation to reality. The agent architecture uses a decoupled representation learning approach based on a predictive Variational Autoencoder, named DVAE, that can fully represent a system modeled as a Markov Decision Process. This formulation allows to learn the visual task with high sample efficiency, requiring far less data than traditional end-to-end Reinforcement Learning methods. This allows us to train a manipulation policy in simulation in less than 12 h.

Furthermore, the decoupled nature of the method and the presence of the dynamics predictor give us the ability to transfer the agent effectively between simulation and reality. Differently from other sim-to-real adaptation works the method proposed in this work is completely unsupervised, is trained online and does not require any target domain knowledge while performing the source domain training. Consequently, it does not require any data collection outside of the experience collected by the RL agent, and as such, it can be applied to difficult exploration problems and tasks for which manual data collection is impractical.

We demonstrated the transfer capabilities of the method *via* sim-to-sim and sim-to-real experiments on an object-pushing robotic setup. The results show how the method can overcome considerable gaps between the characteristics of source and target domain. When the source-target domain gap is small the method can adapt extremely quickly, reducing training time by three to four times. If the reality gap is wider, the method naturally requires more time and data to adapt, but still brings considerable sample-efficiency improvements.

The sim-to-real transfer experiments show how the proposed method offers considerable efficiency improvements especially when looking at the first stages of training. The agent reaches an 80% success rate in just about 500 episodes, half of what is required by a from-scratch training in simulation. It must also be noted how training a policy from scratch in the real would not be as simple as in simulation, the complexity of the real environment would affect the agent performance also in this case. The improvement in real-world training efficiency may thus be greater than what shown.

When looking at the asymptotic performance the method lags when compared with the training from scratch. However this remaining gap in performance can be explained by the more complex nature of the real-world observations, and can potentially be bridged by performing a further finetuning, unfreezing the control policy network after finetuning.

In conclusion, our methodology shows how it is possible to train effectively a manipulation policy such as a robotic object pushing task example with very little real-world data. With just 6 h of real-world experience, the agent learns to solve our object pushing task, directly from visual input.

## Data Availability

The datasets presented in this study can be found in online repositories. The names of the repository/repositories and accession number(s) can be found below: Code will be made available at https://gitlab.com/crzz/dvae_s2r_pushing.

## Footnotes

1 We consider our method to be model-free as we do not use the learned dynamics model to plan trajectories or generate data, but only as a regularization tool. This follows the same convention used in Lee et al. (2020).

## References

[B1] Bousmalis K. Irpan A. Wohlhart P. Bai Y. Kelcey M. Kalakrishnan M. (2018). “Using simulation and domain adaptation to improve efficiency of deep robotic grasping,” in 2018 IEEE international conference on robotics and automation (ICRA) (Brisbane, Australia: IEEE), 4243–4250.

[B2] Bousmalis K. Silberman N. Dohan D. Erhan D. Krishnan D. (2017). “Unsupervised pixel-level domain adaptation with generative adversarial networks,” in Proceedings of the IEEE conference on computer vision and pattern recognition (Honolulu, HI, United States: IEEE), 3722–3731.

[B3] Chua K. Calandra R. McAllister R. Levine S. (2018). “Deep reinforcement learning in a handful of trials using probabilistic dynamics models,” in Advances in Neural Information Processing Systems, NeurIPS 2018. Editors Bengio S. Wallach H. Larochelle H. Grauman K. Cesa-Bianchi N. Garnett R. (Montréal, Canada: Curran Associates, Inc.) 31.

[B5] Ganin Y. Lempitsky V. (2015). “Unsupervised domain adaptation by backpropagation,” in International conference on machine learning (Lille, France: PMLR). 1180–1189.

[B6] Ghosh P. Sajjadi M. S. M. Vergari A. Black M. J. Schölkopf B. (2020). “From variational to deterministic autoencoders,” in 8th International Conference on Learning Representations, ICLR 2020 (Addis Ababa, Ethiopia: OpenReview.net).

[B7] Gupta A. Devin C. Liu Y. Abbeel P. Levine S. (2017). “Learning invariant feature spaces to transfer skills with reinforcement learning,” in International Conference on Learning Representations, ICLR 2017 (Toulon, France: ICLR).

[B8] Haarnoja T. Zhou A. Abbeel P. Levine S. (2018a). “Soft actor-critic: Off-policy maximum entropy deep reinforcement learning with a stochastic actor,” in International conference on machine learning, ICML 2018 (Stockholm, Sweden: PMLR), 1861–1870.

[B9] Haarnoja T. Zhou A. Hartikainen K. Tucker G. Ha S. Tan J. (2018b). Soft actor-critic algorithms and applications. *arXiv preprint arXiv:1812.05905* .

[B11] Hafner D. Lillicrap T. Fischer I. Villegas R. Ha D. Lee H. (2019). “Learning latent dynamics for planning from pixels,” in International conference on machine learning, ICML 2019 (Long Beach, CA, United States: PMLR), 2555–2565.

[B10] Hafner D. Lillicrap T. Ba J. Norouzi M. (2020). “Dream to control: Learning behaviors by latent imagination,” in 8th International Conference on Learning Representations, ICLR 2020 (Addis Ababa, Ethiopia: OpenReview.net).

[B12] Hafner D. Lillicrap T. Norouzi M. Ba J. (2021). “Mastering atari with discrete world models,” in 9th International Conference on Learning Representations, ICLR 2021 (Virtual Event, Austria: OpenReview.net).

[B13] Heiden E. Millard D. Coumans E. Sheng Y. Sukhatme G. S. (2021). “Neuralsim: Augmenting differentiable simulators with neural networks,” in 2021 IEEE International Conference on Robotics and Automation, ICRA 2021 (Xi’an, China: IEEE), 9474–9481.

[B14] Hoffman J. Tzeng E. Park T. Zhu J.-Y. Isola P. Saenko K. (2018). “Cycada: Cycle-consistent adversarial domain adaptation,” in International conference on machine learning, ICML 2018 (Stockholm, Sweden: PMLR), 1989–1998.

[B15] Howard A. Sandler M. Chu G. Chen L.-C. Chen B. Tan M. (2019). “Searching for mobilenetv3,” in Proceedings of the IEEE/CVF international conference on computer vision, ICCV 2019 (Seoul, South Korea), 1314–1324.

[B16] James S. Wohlhart P. Kalakrishnan M. Kalashnikov D. Irpan A. Ibarz J. (2019). “Sim-to-real via sim-to-sim: Data-efficient robotic grasping via randomized-to-canonical adaptation networks,” in Proceedings of the IEEE CVF Conference on Computer Vision and Pattern Recognition, CVPR 2019, 12627–12637.

[B17] Kingma D. P. Ba J. (2015). “Adam: A method for stochastic optimization,” in 3rd International Conference on Learning Representations, ICLR 2015. Editors Bengio Y. LeCun Y. (San Diego, CA, United States).

[B18] Kingma D. P. Welling M. (2014). “Auto-encoding variational bayes,” in 2nd International Conference on Learning Representations, ICLR 2014. Editors Bengio Y. LeCun Y. (Banff, AB, Canada).

[B19] Koenig N. Howard A. (2004). “Design and use paradigms for gazebo, an open-source multi-robot simulator,” in 2004 IEEE/RSJ International Conference on Intelligent Robots and Systems (IROS) (IEEE Cat. No. 04CH37566) (Sendai, Japan: IEEE) 3, 2149–2154.

[B20] Kostrikov I. Yarats D. Fergus R. (2020). Image augmentation is all you need: Regularizing deep reinforcement learning from pixels. *arXiv preprint arXiv:2004.13649* .

[B21] Laskin M. Lee K. Stooke A. Pinto L. Abbeel P. Srinivas A. (2020a). Reinforcement learning with augmented data. Adv. neural Inf. Process. Syst. 33, 19884–19895.

[B49] Laskin M. Srinivas A. Abbeel P. (2020b). “CURL: contrastive unsupervised representations for reinforcement learning,” in Proceedings of the 37th International Conference on Machine Learning, ICML 2020 (Virtual Event: PMLR). Proceed. Machine Learning Res. 119, 5639–5650.

[B22] Lee A. X. Nagabandi A. Abbeel P. Levine S. (2020). “Stochastic latent actor-critic: Deep reinforcement learning with a latent variable model,” in Advances in Neural Information Processing Systems, NeurIPS 2020. Editors Larochelle H. Ranzato M. Hadsell R. Balcan M. Lin H. (Virtual-only: Curran Associates, Inc.) 33, 741–752.

[B23] Long M. Cao Y. Wang J. Jordan M. (2015). “Learning transferable features with deep adaptation networks,” in International conference on machine learning, ICML 2015 (Lille, France: PMLR), 97–105.

[B24] Mnih V. Badia A. P. Mirza M. Graves A. Lillicrap T. Harley T. (2016). “Asynchronous methods for deep reinforcement learning,” in International conference on machine learning, ICML 2016 (PMLR), 1928–1937.

[B25] Mnih V. Kavukcuoglu K. Silver D. Rusu A. A. Veness J. Bellemare M. G. (2015). Human-level control through deep reinforcement learning. nature 518, 529–533. 25719670 10.1038/nature14236

[B26] Muratore F. Gruner T. Wiese F. Belousov B. Gienger M. Peters J. (2021). “Neural posterior domain randomization,” in Conference on Robot Learning, CoRL 2021 (London, United Kingdom: PMLR).

[B27] Nagabandi A. Konolige K. Levine S. Kumar V. (2019). “Deep dynamics models for learning dexterous manipulation,” in 3rd Annual Conference on Robot Learning, CoRL 2019, Proceedings (Osaka, Japan: PMLR), 100, 1101–1112.

[B28] Nair A. Srinivasan P. Blackwell S. Alcicek C. Fearon R. De Maria A. (2015). Massively parallel methods for deep reinforcement learning. *arXiv preprint arXiv:1507.04296* . ICML 2015 Deep Learning Workshop.

[B4] Nvidia (2020). Nvidia isaac sim. Available at: https://developer.nvidia.com/isaac-sim .

[B29] OpenAI Akkaya I. Andrychowicz M. Chociej M. Litwin M. McGrew B. (2019). Solving rubik’s cube with a robot hand. *ArXiv* abs/1910.07113.

[B30] Peng X. B. Andrychowicz M. Zaremba W. Abbeel P. (2017). “Sim-to-real transfer of robotic control with dynamics randomization,” in 2018 IEEE International Conference on Robotics and Automation (ICRA), 1–8.

[B31] Pinto L. Andrychowicz M. Welinder P. Zaremba W. Abbeel P. (2018). “Asymmetric actor critic for image-based robot learning,” in Proceedings of Robotics: Science and Systems, R:SS 2018 (Pittsburgh, Pennsylvania). 10.15607/RSS.2018.XIV.008

[B32] Possas R. Barcelos L. Oliveira R. Fox D. Ramos F. (2020). Online bayessim for combined simulator parameter inference and policy improvement,” in 2020 IEEE/RSJ International Conference on Intelligent Robots and Systems, IROS 2020 (IEEE). IEEE, 5445–5452.

[B33] Raffin A. Hill A. Gleave A. Kanervisto A. Ernestus M. Dormann N. (2021). Stable-baselines3: Reliable reinforcement learning implementations. J. Mach. Learn. Res. 22, 1–8.

[B34] Ramos F. Possas R. Fox D. (2019). “Bayessim: Adaptive domain randomization via probabilistic inference for robotics simulators,” in >Robotics: Science and Systems XV, R:SS 2019. Editors Bicchi A. Kress-Gazit H. Hutchinson S. (Freiburg im Breisgau, Germany). 10.15607/RSS.2019.XV.029

[B35] Rezende D. J. Mohamed S. Wierstra D. (2014). “Stochastic backpropagation and approximate inference in deep generative models,” in Proceedings of the 31st International Conference on Machine Learning. Editors Xing E. P. Jebara T. (Bejing, China: Proceedings of Machine Learning Research) 32, 1278–1286.

[B36] Rudin N. Hoeller D. Reist P. Hutter M. (2021). “Learning to walk in minutes using massively parallel deep reinforcement learning,” in 5th Annual Conference on Robot Learning, CoRL 2021 (London, United Kingdom: Proceedings of Machine Learning Research), 91.

[B37] Ruggiero F. Lippiello V. Siciliano B. (2018). Nonprehensile dynamic manipulation: A survey. IEEE Robotics Automation Lett. 3, 1711–1718. 10.1109/lra.2018.2801939

[B38] Schulman J. Wolski F. Dhariwal P. Radford A. Klimov O. (2017). Proximal policy optimization algorithms. *arXiv preprint arXiv:1707.06347* .

[B39] Srinivas A. Laskin M. Abbeel P. (2020). Curl: Contrastive unsupervised representations for reinforcement learning. *arXiv preprint arXiv:2004.04136* .

[B40] Sun B. Saenko K. (2016). “Deep coral: Correlation alignment for deep domain adaptation,” in Computer Vision—ECCV 2016 Workshops. Editors Hua G. Jégou H. (Amsterdam, Netherlands: Springer International Publishing), 443–450.

[B41] Tobin J. Fong R. Ray A. Schneider J. Zaremba W. Abbeel P. (2017). “Domain randomization for transferring deep neural networks from simulation to the real world,” in 2017 IEEE/RSJ International Conference on Intelligent Robots and Systems (IROS), 23–30.

[B42] Tzeng E. Devin C. Hoffman J. Finn C. Abbeel P. Levine S. (2016). Adapting deep visuomotor representations with weak pairwise constraints (San Francisco, CA, United States: Springer International Publishing), 688–703. 10.1007/978-3-030-43089-4_44

[B43] Tzeng E. Devin C. Hoffman J. Finn C. Peng X. Levine S. (2015). Towards adapting deep visuomotor representations from simulated to real environments. *CoRR* arXiv preprint arXiv:1511.07111.

[B44] Tzeng E. Hoffman J. Saenko K. Darrell T. (2017). “Adversarial discriminative domain adaptation,” in Proceedings of the IEEE conference on computer vision and pattern Recognition, CVPR 2017 (Honolulu, HI, United States).

[B45] Tzeng E. Hoffman J. Zhang N. Saenko K. Darrell T. (2014). Deep domain confusion: Maximizing for domain invariance. *CoRR* arXiv preprint arXiv:1412.3474.

[B46] Unity (2020). Unity. Available at: https://unity.com/solutions/automotive-transportation-manufacturing/robotics .

[B47] Yarats D. Fergus R. Lazaric A. Pinto L. (2022). “Mastering visual continuous control: Improved data-augmented reinforcement learning,” in The Tenth International Conference on Learning Representations, ICLR 2022 (Virtual Event: OpenReview.net).

[B50] Yarats D. Kostrikov I. Fergus R. (2021a). “Image augmentation is all you need: Regularizing deep reinforcement learning from pixels,” in 9th International Conference on Learning Representations, ICLR 2021, Virtual Event, Austria, May 3–7, 2021 (OpenReview.net).

[B48] Yarats D. Zhang A. Kostrikov I. Amos B. Pineau J. Fergus R. (2021b). “Improving sample efficiency in model-free reinforcement learning from images,” in Proceedings of the Thirty-Fifth AAAI Conference on Artificial Intelligence, AAAI 2021 (Held Virtually: AAAI Press), 10674–10681.

